# Penicillin resistance and serotype distribution of *Streptococcus pneumoniae* in Ghanaian children less than six years of age

**DOI:** 10.1186/1471-2334-13-490

**Published:** 2013-10-22

**Authors:** Nicholas TKD Dayie, Reuben E Arhin, Mercy J Newman, Anders Dalsgaard, Magne Bisgaard, Niels Frimodt-Møller, Hans-Christian Slotved

**Affiliations:** 1 Department of Microbiology, University of Ghana Medical School, Accra, Ghana; 2 Department of Veterinary Disease Biology, Faculty of Health and Medical Sciences, University of Copenhagen, Copenhagen, Denmark; 3 Department of Clinical Microbiology, University Hospital, Hvidovre, Copenhagen, Denmark; 4 Department of Microbiological Surveillance and Research, Statens Serum Institut, Copenhagen, Denmark

## Abstract

**Background:**

The objective of this study was to determine the prevalence of nasopharyngeal carriage, serotype distribution, and penicillin resistance of *Streptococcus pneumoniae* in children ≤6 years of age in Ghana.

**Methods:**

A cross-sectional study was carried out on a cluster-randomized sample of children ≤6 years of age attending nurseries and kindergartens in Accra and Tamale, Ghana. Basic data on age, sex and exposure to antimicrobials in the previous month were collected on all study subjects. Nasopharyngeal swabs were obtained from participants and all pneumococcal isolates were characterized by serotyping and their penicillin resistance determined.

**Results:**

The overall prevalence of pneumococcal carriage among the children was 34% in Accra and 31% in Tamale. The predominant serotypes were 19F, 6B, 23F, and 6A with 23% of the isolates being non-typable in Accra and 12% in Tamale. Only two isolates (serotypes 19F and 6B) from Tamale had a MIC >2 μg/ml and were classified as fully penicillin resistant with 45% of the isolates having intermediate resistance.

**Conclusions:**

These findings indicate that the 13-valent pneumococcal conjugate vaccine (PCV-13) recently introduced in Ghana will cover 48% and 51% of the serotypes identified in Accra and Tamale, respectively. The 23-valent pneumococcal polysaccharide vaccine (PPV-23) will cover 54% of all serotypes detected. The two penicillin resistant isolates (MIC 32 μg/ml) were serotypes included in both PCV-13 and PPV-23. A nationwide monitoring system of penicillin susceptibility patterns and pneumococcal serotypes is recommended.

## Background

*Streptococcus pneumoniae* (pneumococcus) is an important cause of morbidity and mortality among children less than five years of age, patients with debilitating diseases, the elderly (≥65 years) and immunocompromised individuals [1-3]. Globally, around two million children die every year because of pneumococcal pneumonia and meningitis, mainly in developing countries [4,5]. In recent years, there has been an increasing focus on the introduction of pneumococcal conjugate vaccines (PCV) in resource-limited countries [6-8]. The introduction of PCV is expected to reduce the burden of invasive pneumococcal diseases (IPD) in children as well as the prevalence of drug resistant *S. pneumoniae*[7].

Nasopharyngeal carriage of *S. pneumoniae* always precedes disease and serves as a reservoir for the transmission of the pathogen [9]. Although there are more than 90 known pneumococcal serotypes, about 20 serotypes account for over 75% of the IPD cases [10]. Each serotype differs in prevalence, age group infected, geographical distribution, and antimicrobial resistance patterns [11-15]. Local knowledge of circulating serotypes and their antimicrobial susceptibility profiles is therefore imperative for the development of effective vaccine strategies and treatment protocols [7,16]. Several vaccines have been developed including a 23-valent polysaccharide vaccine (PPV-23, Pneumovax®, Merck) used to immunize the elderly and children over two years of age. For children under two years of age, a 10-valent pneumococcal *Haemophilus influenzae* conjugate vaccine (PCV-10, GlaxoSmithKline) and a 13-valent pneumococcal conjugate vaccine (PCV-13, PrevnarTM, Pfizer Vaccines) are available.

In Ghana, three studies have assessed the carriage rate of *S. pneumoniae*[17-19] but none have reported the prevalence of both serotypes and penicillin susceptibility in isolates from healthy children below the age of 6 years. In this study, we aimed to generate baseline data to inform vaccine policy and treatment strategies in Ghana. The objective of the study was, therefore, to determine the prevalence of pneumococcal carriage, penicillin resistance and the serotype distribution of *S. pneumoniae* in children ≤6 years attending nurseries and kindergartens in two cities in Ghana; Accra in the south and Tamale in the north.

## Methods

### Study sites

The cities of Accra and Tamale are located in two geographical areas in Ghana with distinct climates. Accra is the capital city of Ghana with a costal climate, and warm and humid weather conditions. Tamale is the regional capital of the Northern Region of Ghana. It is situated close to the Sahara, and has a hotter, drier climate characterized by dry northeasterly winds (Harmattan) [19], http://www.ghanadistricts.com/districts].

### Sampling and study design

Random cluster sampling was used to select participants. Lists of nurseries (children aged <48 months) and kindergartens (children aged 48-72 months) were obtained from the Education Service of the Accra Metropolitan Assembly and the Tamale Metropolitan Assembly. A total of 11 nurseries and kindergartens in Accra and seven in Tamale were randomly chosen from the lists provided. All children attending the selected nurseries and kindergartens were eligible to be included in the study. Children with upper respiratory tract infections at the time of sample collection and who had been treated with antimicrobials in the previous month were excluded. Although the study sites were mainly situated within the cities, some of the nurseries and kindergartens were located in slums and in addition, children may have come from the surrounding villages to attend school in the cities.

### Specimen collection

Nasopharyngeal specimens were collected using a WHO recommended methodology [20]. From March to July 2011, nylon-tipped pediatric sized swabs (Microrheologics, Brescia, Italy) were used to collect nasopharyngeal specimens. A total of 848 swab samples were obtained from individual children in Accra (421) and Tamale (427). The swab specimens were placed in a labeled vial containing 1 ml skim milk-tryptone-glucose-glycerin (STGG) medium and transported on ice to the laboratory within 3 h [20]. Depending on the time of the day samples were either processed within 24 h or stored at −80°C. All *S. pneumoniae* isolates were stored in STGG medium at −80°C until air-lifted on dry ice to Statens Serum Institut, Copenhagen, Denmark for further characterization.

### Characterization of *S. pneumoniae*

A 10-μl loop subsample of each specimen was cultured on 5% sheep blood agar plates and incubated overnight at 37°C in 5% CO_2_. All alpha-hemolytic organisms were subjected to Gram staining, optochin susceptibility and bile solubility tests [20]. Isolates that were optochin sensitive and/or bile soluble were identified as *S. pneumoniae*. These isolates were serotyped/grouped by the pneumotest latex agglutination kit (SSI Diagnostica, Hillerød, Denmark) and results confirmed by the Quellung reaction using serotype specific antisera (SSI Diagnostica) [21]. All specimens were screened for multiple serotypes. Briefly, the specimens were cultured in an enrichment broth (Serum broth, SSI Diagnostica) and then screened using a pneumotest latex agglutination kit (SSI Diagnostica) [21]. Where multiple serotypes were observed, they were isolated and serotyped as described above.

Penicillin resistance was initially determined by agar-disc diffusion using 1 μg oxacillin discs (Rosco Company, Denmark). Minimum inhibitory concentrations (MICs) for all oxacillin resistant isolates were determined using penicillin G MIC strips (Liofilchem, Italy). Penicillin susceptibility was defined as susceptible (MIC ≤0.06 μg/ml), intermediate (>0.06-2 μg/ml) and resistant (>2 μg/ml) according to the European Committee on Antimicrobial Susceptibility Testing (EUCAST) guidelines with *S. pneumoniae* ATCC 49619 used as a control (EUCAST Clinical Breakpoint Table v. 2.0, valid from 2012-01-01).

### Ethical approval

Ethical approval for the study was obtained from the University of Ghana Medical School (MS-Et/M.5-P.5.4/2010-11) and permission to conduct the study was obtained from the Ministry of Education and Health. Informed consent was provided by the parents of children participating in the study.

### Data analysis

Data were analyzed using Graph Pad Prism version 5 for descriptive statistical analysis.

## Results

### Serotype prevalence and distribution

Nasopharyngeal swabs were collected from a total of 848 children ≤6 years of age (52% male). The average age of the children in both sites was similar (4.1 years in Accra and 4.0 years in Tamale). In total 274 children (32%, 95% CI 29%-36%) were found to carry pneumococci, with similar rates found in Accra and Tamale (Table 1). Because the study subjects were from nurseries and kindergartens relatively few children aged 0-11 months (n = 7) and 12-23 months (n = 73) were included (Table 1).

**Table 1:** **Carriage rate of *S. pneumoniae*, by age group, in children ≤6 years attending nurseries and kindergartens in Accra and Tamale, Ghana**

**Age group (months)**	**Accra**			**Carriage of two distinct serotypesTamale**		
	**Total number of children**	**Number of children with carriage of *S. pneumoniae *** **(%) [95% CI]**	**Number of children with multiple serotypes (%)**	**Total number of children**	**Number of children with carriage of *S. pneumoniae *** **(%) [95% CI]**	**Number of children with multiple serotypes (%)**
0-11	4	4 (100)	2 (50)	3	2 (67)	0
		(40-100%)			(13-98%)	
12-23	33	20 (61)	1 (3)	40	16 (40)	0
		(42-77%)			(25-57%)	
24-35	71	30 (42)	3 (4)	66	21 (32)	0
		(31-55%)			(21-44%)	
36-47	135	46 (34)	4 (3)	132	43 (33)	2 (1)
		(26-43%)			(25-41%)	
48-59	158	35 (22)	0	184	48 (26)	2 (1)
		(16-29%)			(20-33%)	
Unknown	20	8 (40)	0	2	1 (50)	0
		(19-64%)			(1-99%)	
Total	421	143 (34)	10 (2)	427	131 (31)	4 (1)
		(30-39%)			(26-35%)	

**Figure 1:**
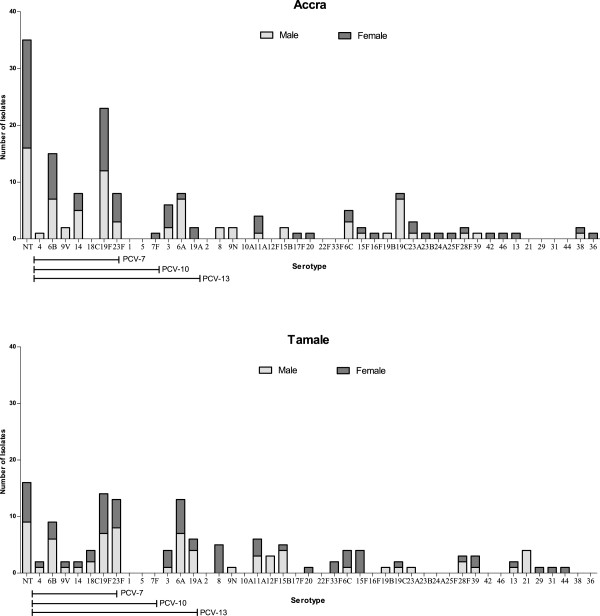
**Serotype distribution of *S. pneumoniae*, by gender, in children ≤6 years of age attending nurseries and kindergartens in Accra and Tamale, Ghana.** The serotypes are listed on the X-axis, starting with the NT, and followed by the serotypes covered by PCV-7, PCV-10 and PCV-13. The serotypes covered by PPV-23 are listed consecutively from serotype 4 to serotype 33F (except for serotype 6A, which is not included in PPV-23).

Carriage of two distinct serotypes occurred in 5% of children (n = 14), giving a total of 288 serotypes isolated from the 274 children sampled. Eighteen percent of all isolates (51/288) were non-typable. The most common serotypes identified were 19F, 6B, 23F, and 6A (Figure 1 and Table 2). The predominant serotype in Accra was 19F (16%), followed by 6B, 19C, 23F, 6A, 6C, and 14 (Figure 1 and Table 2). In Tamale, the predominant serotype was also 19°F (11%), followed by 23F, 6A, 6B, 8, 11A, and 15B (Figure 1 and Table 2). In both Accra and Tamale the prevalence of carriage decreased with age (Table 1).

**Table 2:** **Combined distribution of *S. pneumoniae *** **serotypes and vaccine coverage in children ≤6 years attending nurseries and kindergartens in Accra and Tamale, Ghana**

	**Age of children (Accra/Tamale), months**						**Total number Accra/Tamale**	**Serotype included in vaccine**
**Serotype**	**0-11**	**12-23**	**24-35**	**36-47**	**48-59**	**Unknown**	**All age groups**	
NT	0/1	0/2	9/0	14/8	9/5	3/0	35/16	
4	0/0	1/0	0/0	0/2	0/0	0/0	1/2	PCV-10, PCV-13, PPV-23
6B	1/0	0/3	3/0	2/2	8/4	1/0	15/9	PCV-10, PCV-13, PPV-23
9V	0/0	0/0	1/1	1/0	0/1	0/0	2/2	PCV-10, PCV-13, PPV-23
14	0/0	3/0	2/2	1/0	1/1	1/0	8/2	PCV-10, PCV-13, PPV-23
18C	0/0	0/0	0/0	0/2	0/2	0/0	0/4	PCV-10, PCV-13, PPV-23
19F	1/1	12/5	4/4	3/3	2/1	1/0	23/14	PCV-10, PCV-13, PPV-23
23F	2/0	1/2	0/0	5/3	0/8	0/0	8/13	PCV-10, PCV-13, PPV-23
1	0/0	0/0	0/0	0/0	0/0	0/0	0/0	PCV-10, PCV-13, PPV-23
5	0/0	0/0	0/0	0/0	0/0	0/0	0/0	PCV-10, PCV-13, PPV-23
7F	0/0	0/0	0/0	0/0	1/0	0/0	1/0	PCV-10, PCV-13, PPV-23
3	0/0	1/0	1/2	3/0	1/2	0/0	6/4	PCV-13, PPV-23
6A	1/0	0/1	3/5	4/1	0/6	0/0	8/13	PCV-13
19A	0/0	0/0	1/1	0/2	1/2	0/1	2/6	PCV-13, PPV-23
2	0/0	0/0	0/0	0/0	0/0	0/0	0/0	PPV-23
8	0/0	1/1	1/0	0/4	0/0	0/0	2/5	PPV-23
9N	0/0	0/0	0/0	0/0	2/1	0/0	2/1	PPV-23
10A	0/0	0/0	0/0	0/0	0/0	0/0	0/0	PPV-23
11A	0/0	0/0	0/2	3/3	1/1	0/0	4/6	PPV-23
12F	0/0	0/0	0/0	0/1	0/2	0/0	0/3	PPV-23
15B	0/0	0/0	0/2	2/2	0/1	0/0	2/5	PPV-23
17F	0/0	0/0	0/0	1/0	0/0	0/0	1/0	PPV-23
20	0/0	0/0	0/0	0/0	1/1	0/0	1/1	PPV-23
22F	0/0	0/0	0/0	0/0	0/0	0/0	0/0	PPV-23
33F	0/0	0/0	0/0	0/2	0/0	0/0	0/2	PPV-23
Other	1/0	2/2	8/3	11/10	8/12	2/0	32/27	Non-vaccine serotypes

### Antimicrobial resistance

In Accra, 66 of *S. pneumoniae* isolates were oxacillin resistant while in Tamale 62 of the isolates were resistant. The oxacillin resistant isolates, plus 13 isolates not tested for oxacillin were further tested using penicillin G MIC strips. In total 130/288 isolates (45%) had intermediate resistance to penicillin and two (from Tamale) were penicillin resistant (MIC 32 μg/ml) (Table 3).

**Table 3:** **Distribution of *S. pneumoniae *** **isolates with intermediate and full penicillin resistance by vaccine coverage**

**Vaccine coverage**	**Number of isolates**		**Penicillin**		**Penicillin**	
			**(MIC 0.06-2)**		**(MIC > 2)**	
	**Accra**	**Tamale**	**Accra**	**Tamale**	**Accra**	**Tamale**
	**(N = 153)**	**(N = 135)**	**(N = 67)**	**(N = 63)**	**(N = 0)**	**(N = 2)**
Non-typable	35	16	10	7	0	0
PCV-7	57	46	30	19	0	1
PCV-10	58	46	30	19	0	1
PCV-13	74	69	40	32	0	2
PPV-23	78	79	40	39	0	2
NON-PCV	79	66	27	31	0	0

### Vaccine coverage

Our serotyping results indicate that PCV-13 covers approximately half of the serotypes carried by healthy children ≤6 years of age in our study (48% in Accra and 51% in Tamale), while PPV-23 provides coverage for 55% of all isolates in our sample (Table 2). In total, PCV-13 covers 55% of the isolates with intermediate penicillin resistance, compared with PPV-23 which covers 61%. The coverage rates were similar in both Accra and Tamale. The two isolates that were fully penicillin resistant were serotypes 19A and 6B, both of which were covered by PCV-13 and PPV-23. The majority (80%) of serotypes seen in children under 24 months of age were covered by PCV-13 (31/39). A higher proportion of isolates from children aged 3-6 years were not covered by PCV-13 (Table 2).

## Discussion

Recently, the GAVI Alliance (formerly the Global Alliance for Vaccines and Immunization), a partnership of public and private sector organizations dedicated to “immunization for all” (http://www.gavialliance.org), started the introduction of pneumococcal vaccines in developing countries, especially in Africa [8], and in May 2012 PCV-13 was introduced in Ghana. Studies on the carriage rates of *S*. *pneumoniae* in healthy subjects should be conducted before the introduction of conjugate pneumococcal vaccination campaigns to provide baseline information on vaccine coverage and to allow identification of changes to the serotype distributions resulting from pneumococcal serotype replacement [8].

Our study provides the first large data set on *S*. *pneumoniae* carriage and serotype distribution in healthy Ghanaian children. Carriage prevalence was 31%-34% in our study compared with the 15% previously reported in Ghana [18]. This difference might be due to the age differences between our study subjects (≤6 years of age) and those of the previous study, which included children ≤13 years of age [18]. The overall carriage in our study was relatively low when compared with prevalence rates reported in other African countries; 22%-60% in Kenya [22,23], 62% in Uganda [24], 90% in Gambia [25] and 35% in Tanzania [26]. This may be due to the low numbers of children below the age of two years included in this study (Table 1). As our data on pneumococcal carriage were collected before the introduction of PCV-13, they can serve as a baseline to measure possible future serotype replacement associated with the introduction of PCV-13 [8].

Penicillin has been the drug of choice worldwide for the treatment of pneumococcal infections. However, penicillin resistant strains have emerged, resulting in a shift to the use of other drugs, e.g. cefotaxime, chloramphenicol and erythromycin [18]. In an earlier carriage study from the Ashanti region of Ghana, 49% of children were found to carry *S. pneumoniae* and 39% of isolates showed intermediate penicillin resistance [17]. Holliman et al. [27] described the history of *S. pneumoniae* penicillin resistance in Ghana and found that overall around 12% of isolates tested were intermediate resistant with the exception of a study in 1996 that reported 31% of isolates to be resistant [28]. However, this high rate of resistance was suspected to be due to the methodology used in the specific study [27,28]. In a recent study conducted in the Ashanti region from 2008 to 2010, 99% of 91 invasive *S. pneumoniae* isolates were penicillin sensitive [29]. In our study, two isolates were found to be fully penicillin resistant and 45% (n = 130) showed intermediate penicillin resistance. These data suggest that intermediate resistance to penicillin has increased among *S. pneumoniae* in Ghana during the last 5-10 years and that the prevalence of penicillin resistance is in line with the situation in other African countries. Despite a possible increase in intermediate penicillin resistance, and depending on the site of infection, penicillin can still be used for the treatment of pneumococcal infections in Ghana.

The *S. pneumoniae* serotypes found show that PPV-23 covers 55% of serotypes while PCV-10 and PCV-13 cover 40% and 50% of serotypes, respectively. As PCV-13 is currently used nationwide in Ghana to vaccinate children ≤5 years with a vaccination schedule at 6, 10 and 14 weeks, there might be a high risk of replacement with serotypes not covered by PCV-13. In addition, a high proportion of non-vaccine serotypes exhibited intermediate resistance to penicillin. It is therefore imperative that nasopharyngeal carriage and penicillin resistance of *S. pneumoniae* be monitored regularly in children [8]. Prior to our study, limited data were available on serotype distribution among *S. pneumoniae* in Ghana [18,19]. Thus, contrary to recommendations [7,8] recent surveillance data on prevalent serotypes were not available in Ghana when PCV-13 was introduced in May 2012. Our finding that only 50% of the serotypes found are included in PCV-13 highlights the importance of conducting such carriage studies before the introduction of any vaccine.

In this study we focused on children from nurseries and kindergarten, and as a result, very few children <11 months of age were included. This is a limitation of the study, as other studies in Africa have shown a very high carriage rates in this age group [30]. We chose to study children attending nurseries and kindergartens as the length of time and close physical proximity to other children in such locations constitutes an optimal environment for horizontal spread of pneumococci [31]. These sites act like reservoirs of different serotypes, which can then easily spread to the surrounding community. It is therefore of interest to see which non-vaccine serotypes might take over after vaccination [32]. Although we only recruited children from two cities in Ghana, we are of the opinion that the data on carriage rates are representative of other regions of Ghana as we did not observe large differences between the carriage rates of the two sites, Accra and Tamale, even though they are geographically apart and have different climates. In addition, we did not observe any marked differences in serotype distribution between the two study sites (Figure 1, Tables 1 and 2).

## Conclusions

The increase in intermediate penicillin resistance in *S. pneumoniae* in Ghana is of some concern and should be monitored carefully. The current study shows that PCV-13, introduced in Ghana in 2012, covers only 50% of the prevalent serotypes found in healthy child carriers and that the risk for serotype replacement is high. Thus, regular monitoring of pneumococcal carriage is essential to identify newly dominant serotypes for future vaccine formulations and to determine prevalence of antimicrobial resistance. Our study provides the most recent baseline data for pneumococcal serotypes and penicillin resistance in Ghanaian children.

## Competing interests

The authors declare that they have no competing interests.

## Authors’ contributions

NTKDD, AD, NFM, HCS conceived and designed the study. NTKDD, REA, MJN, AD, MB, NFM, HCS contributed to the protocol writing. NTKDD, REA collected the clinical samples. NTKDD, REA, HCS conducted the laboratory assays. NTKDD, AD, NFM, HCS analyzed the data. NTKDD, AD, NFM, HCS drafted the manuscript. NTKDD, REA, MJN, AD, MB, NFM, HCS reviewed the data and critically revised the manuscript. All authors have read and approved the final manuscript.

## Pre-publication history

The pre-publication history for this paper can be accessed here:

http://www.biomedcentral.com/1471-2334/13/490/prepub
